# Development of CNN models for the enteral feeding tube positioning assessment on a small scale data set

**DOI:** 10.1186/s12880-022-00766-w

**Published:** 2022-03-22

**Authors:** Gongbo Liang, Halemane Ganesh, Dylan Steffe, Liangliang Liu, Nathan Jacobs, Jie Zhang

**Affiliations:** 1 Eastern Kentucky University, Richmond, KY USA; 2 University of Kentucky, Lexington, KY USA; 3 Henan Agricultural University, Zhengzhou, China

**Keywords:** Weakly supervised, Pre-training, Annotation-efficient modeling

## Abstract

**Background:**

Enteral nutrition through feeding tubes serves as the primary method of nutritional supplementation for patients unable to feed themselves. Plain radiographs are routinely used to confirm the position of the Nasoenteric feeding tubes the following insertion and before the commencement of tube feeds. Convolutional neural networks (CNNs) have shown encouraging results in assisting the tube positioning assessment. However, robust CNNs are often trained using large amounts of manually annotated data, which challenges applying CNNs on enteral feeding tube positioning assessment.

**Method:**

We build a CNN model for feeding tube positioning assessment by pre-training the model under a weakly supervised fashion on large quantities of radiographs. Since most of the model was pre-trained, a small amount of labeled data is needed when fine-tuning the model for tube positioning assessment. We demonstrate the proposed method using a small dataset with 175 radiographs.

**Result:**

The experimental result shows that the proposed model improves the area under the receiver operating characteristic curve (AUC) by up to 35.71% , from 0.56 to 0.76, and 14.49% on the accuracy, from 0.69 to 0.79 when compared with the no pre-trained method. The proposed method also has up to 40% less error when estimating its prediction confidence.

**Conclusion:**

Our evaluation results show that the proposed model has a high prediction accuracy and a more accurate estimated prediction confidence when compared to the no pre-trained model and other baseline models. The proposed method can be potentially used for assessing the enteral tube positioning. It also provides a strong baseline for future studies.

## Background

Enteral nutrition through feeding tubes serves as the primary method of nutritional supplementation for patients unable to feed themselves. The position assessment of the Nasoenteric feeding tubes is essential following insertion and before the commencement of tube feeds to avoid potential complications [1]. A plain radiograph is typically performed to confirm the placement of the feeding tube [2]. The feeding tube position assessment is straightforward but costly and time-consuming. Timely interpretation of the radiographs remains a challenge affecting clinical decision to start tube feeds.

Recently, artificial neural networks (ANNs) have shown great potential to be an effective tool to detect and diagnose medical problems [3–5]. As a data-driven approach in the concept of supervised learning, ANNs learn data features automatically from the given training set of samples and labels [6–8]. Deep convolutional neural networks (CNNs), as a subset of ANNs, have shown promising results in various medical imaging analysis tasks [9–11]. For instance, Esteva et al. applied CNN models to dermatoscopy images for skin cancer diagnosis and achieved performance on par with all participated human dermatologists. Ribli et al. used an rCNN-based [12] method for 2D mammograms classification that achieved 0.95 AUC for breast tumor classification [13]. Ying et al. proposed a cross-modality ANN model for Alzheimer’s disease (AD) diagnosis that used a CNN to evaluate head MRIs and a multilayer perceptron (MLP) model to analysis the single-nucleotide polymorphisms (SNPs) information from Genome-wide association study (GWAS) [14]. Their proposed model achieved a 0.935 AUC on AD diagnosis. However, robust CNNs commonly require large amounts of manually annotated data for training [15–18], such as ImageNet [19], which contains over one million images with labels. The prohibitively high annotation cost often presents a barrier to adopting modern CNN techniques in the medical imaging analysis tasks [20–23].

Transfer learning and pre-training are widely used in the medical imaging analysis that enables the training of CNN on small datasets [24–26]. In general, this includes three steps: first, a CNN model is pre-trained on a large dataset, such as ImageNet, for classification tasks; second, the feature extractor (i.e., the convolutional layers) of the pre-trained model is selected to be used as the backbone building block of another CNN model; third, the newly built CNN model is fine-tuned on a small medical imaging dataset for the specific purpose. Transfer learning from the ImageNet dataset to medical datasets has shown a promising result in improving the network performance on small medical datasets [24, 26–28], such a technique is also used to build enteral feeding tube positioning assessment models. For instance, Singh et al. transferred the ImageNet pre-trained model to the enteral feeding tube positioning assessment task and significantly improved the small training set [24]. However, an obvious domain gap exists between ImageNet images (i.e., natural images) and medical images, raising concerns about such a method [29].

We propose to use a novel pre-training method [30] to train CNN models on a small datasets for enteral feeding tube positioning assessment. Different from the early study [24], our method uses radiological imaging reports as weak supervision to pre-train the feature extractor on a large radiograph dataset before transfer learning is applied to build the feeding tube positioning assessment model. Radiological imaging reports are routinely collected in clinical practice and readily available in the medical record system. No additional manual labeling is required for pre-training of the proposed method. More importantly, the radiographs for pre-training are directly relevant to the enteral feeding tube positioning assessment task, mitigating the domain gap between natural imaging and radiographs posed by pre-training on the ImageNet dataset.

**Fig. 1:**
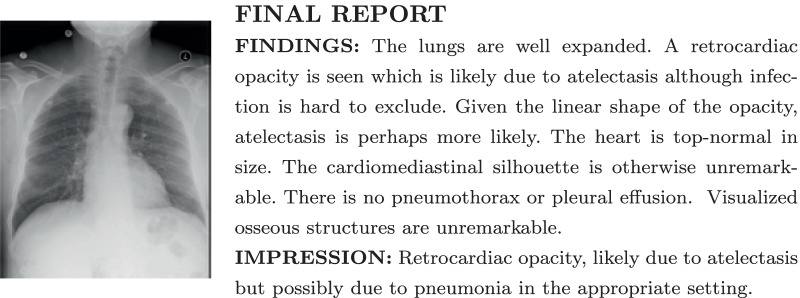
An example of a radiograph (left) with the radiology report (right) from the MIMIC-CXR dataset [31]

## Method

### Model development

We previously developed a general pre-training strategy, which used the radiology reports as weak supervision to pre-train a CNN model that improves the model performance on a given task [30]. This work extended the previous method to build an automatic enteral feeding tube positioning assessment network using a small training dataset. We assume two datasets, $X_P$ and $X_L$ ($|X_P| \gg |X_L|$), exist, where $X_P$ contains paired of radiographs and associated radiology reports and $X_L$ consists of labeled radiographs for enteral feeding tube positioning assessment. Our proposed network pre-trained the feature extractor of the enteral feeding tube positioning assessment model on $X_P$ directly without requiring manually annotated labels. The feature extractor, then, was fine-tuned on $X_L$ for the enteral feeding tube positioning assessment. Figure 1 shows an example of a radiograph and the corresponding radiology report.

#### Pre-training feature extractor via radiograph-report matching

**Fig. 2:**
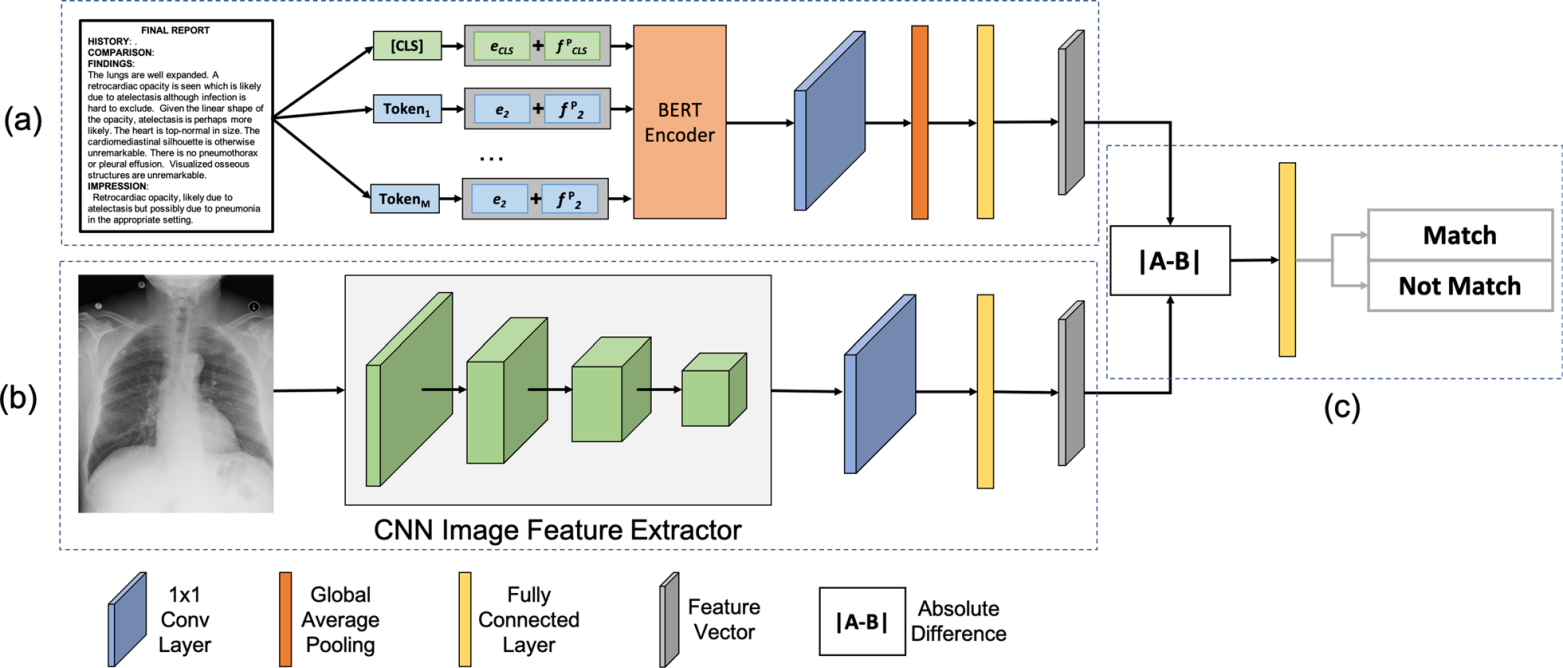
Illustration of the weakly supervised pre-training approach

We pre-trained the feature extractor of the enteral feeding tube positioning assessment model through a radiograph-report matching network (Fig. 2), containing a textual report processing branch (Fig. 2a), a radiograph processing branch (Fig. 2b), and a contrastive learning module (Fig. 2c). The two branches worked simultaneously in parallel. The network took a radiology report and radiograph pair as input and predicted whether they were a natural match. Since label (i.e., match or don’t match) is known, no manual annotation will be required. This weakly supervised pre-training approach transfers the rich information in reports to the radiograph feature extractor without requiring manually labeled data.

Specifically, the textual report processing branch (Fig. 2a) took a radiology report as input and (1) passed the report through a pre-trained BERT (Bidirectional Encoder Representations from Transformers) [32] encoder and a $1\times 1$ convolutional (Conv) layer to convert the natural language in the report to numerical embeddings, i.e., a sequence of numbers that can be processed by computer algorithms, (2) reduced the dimensionality of the embeddings by applying a global average pooling (GAP) operation, and (3) projected the embeddings to a latent feature space by a fully connected (FC) layer. The output of the textual report processing branch was a feature vector that represented the report in latent space. Meanwhile, the radiograph processing branch (Fig. 2b) took a radiograph as input and passed it through a ResNet-18 [15] feature extractor. The generated feature map was then passed through a Conv layer with $1\times 1$ kernels to transfer the pre-trained features to task-specific features. After that, an FC layer was used to embed the radiograph feature map to the latent space, which is the same as the textual report features. The output of the radiograph processing branch was a feature vector in the latent space that represented the input radiograph. Next, the radiograph-report matching network was trained in a contrastive manner via the contrastive learning module (Fig. 2c). A shallow CNN classifier was added on top of the two branches that takes the absolute difference between the two feature vectors as input and ouputs whether the two feature vectors belonged to the same example.

Mathematically, the radiograph-report matching network could be written as:

1 $$\begin{aligned} h_{\theta _p}(x^{i}) = h_{\theta _{cls}}(|h_{\theta _t}(x_t^{i}) - h_{\theta _r}(x_r^{i})|). \end{aligned}$$

where $x^{i}=\{x_t^{i}, x_r^{i}\}$ was a pair of a textual radiology report, $x_t^{i}$, and a radiograph, $x_r^{i}$ from $X_P$. Note that $x_t^{i}$ and $x_r^{i}$ may or may not match. The network $h_{\theta _p}(\cdot )$ predicted the probability of the input pair being a natural match. The $h_{\theta _{cls}}(\cdot )$ was the contrastive learning module, $h_{\theta _t}(\cdot )$ was the textual report processing branch, and $h_{\theta _r}(\cdot )$ was the radiograph processing branch. Binary cross-entropy loss was used to train the text-image matching network.

The input of the radiograph-report matching network was a radiology report and radiograph pair. A label was naturally assigned to each radiograph-report shwoing whether are from the same imaging event. A true pair meant the report describes the radiograph naturally; otherwise, it was a false pair.

#### CNN for enteral feeding tube Positioning assessment

**Fig. 3:**
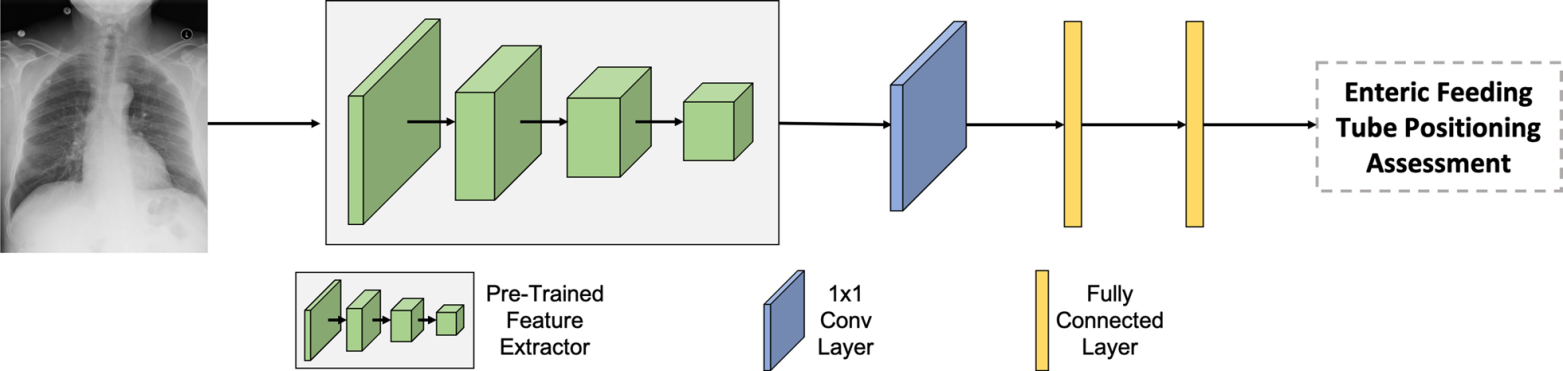
The enteral feeding tube positioning assessment network

The enteral feeding tube positioning assessment model was trained by fine-tuning the feature extractor in the radiograph processing branch, $h_{\theta _r}(\cdot )$, of the pre-trained network. The process was straightforward and illustrated in Fig. 3. The Conv layers in $h_{\theta _r}(\cdot )$ were used as the feature extractor in the enteral feeding tube positioning assessment model. A Conv layer and two FC layers were added on top of the feature extractor to build the classification model for the enteral feeding tube positioning assessment network, $h_{\theta }(\cdot )$. The $h_{\theta }(\cdot )$ took radiographs from $X_L$ and predicted the probability of the enteral feeding tube positions being satisfied. Since the feature extractor was pre-trained using a larger dataset set from the same domain, we only need to optimize the $h_{\theta }(\cdot )$ from scratch that may use significantly reduce the need for the total number of training instances.

### Enteral feeding tube positioning dataset

A dataset containing plain radiographs of 175 patients was retrospectively retrieved at a comprehensive tertiary academic medical center. All the images were inspected by a board-certified abdominal radiologist with more than 10 years of experience and a trainee. The dataset included 63 images where the enteral feeding tube positioning was unsatisfactory, and 112 images with a satisfying position. This retrospective study was approved by the Institutional Review Boards of the University of Kentucky.

The pixel values of radiographs were converted to the range of 0-255 using a window of 0-2750. The images were resized to $256\times 256$ and equally split into five folds for a fivefold cross-testing. Real-time data augmentation for the combination of a random horizontal flip and rotation between 0 and 20 degrees was applied to the training data.

### Model evaluation

#### Compared models

We compared the proposed model with the CNN models trained using four different pre-training strategies: (a) a CNN model without pre-training (denoted as *No Pre-Train*), (b) a CNN model pre-trained on ImageNet [19] (denoted as *ImageNet*), (c) a CNN model pre-trained using *Compare to Learning* [29], a state-of-the-art self-supervised pre-training model for 2D medical images (denoted as *C2L*), and (d) a true random CNN model (denoted as *Random*). All models have the same architecture.

The *No Pre-Train* model was a typical CNN model trained using the enteral feeding tube dataset only. No pre-training strategy was applied. All the weights of this model were randomly initialized before the training.

The *ImageNet* model was a CNN model that pre-trained on the ImageNet dataset, a natural imaging dataset containing over one million images of 1000 classes. Such a pre-training method is well-accepted and widely used in the medical imaging domain [26–28], which was also used in [24], an early study of enteral feeding tube positioning assessment using CNN models. The model was trained on the ImageNet dataset for a classification task and was fine-tuned using the enteral feeding tube dataset under the same approach of Sect. 2.1.2.

The *C2L* model was pre-trained using *Comparing to Learn* [29] on the MIMIC-CXR dataset [31] that was a self-supervised, pre-training method that was proposed for medical imaging analysis. The method pre-trained a feature extractor on MIMIC-CXR, containing 227, 835 radiographic studies of 64, 588 patients that including 368, 948 chest radiographs and the associated radiology reports. The model, then, was fine-tuned using the enteral feeding tube dataset under the same approach of Sect. 2.1.2.

The *Random* model was a CNN model with randomly initialized weights. The model was not trained with any data samples. The model performs random guessing for any input examples.

The proposed model was pre-trained using [30] that was proposed by our previous study. Specifically, the feature extractor of the proposed method was pre-trained on MIMIC-CXR for radiograph-report matching tasks. The detailed pre-training setup of the proposed method was described in [30]. After the feature extractor was pre-trained, the network was fine-tuned using the enteral feeding tube dataset under the same approach of Sect. 2.1.2. No radiology reports were needed for fine-tuning or testing the enteral feeding tube positioning assessment model.

All the compared models were trained for five trials with a fivefold cross-testing strategy. We used three folds for training, one for validation, and one for testing. We repeated this process until all folds were tested. The validation fold is used to select the best checkpoint of the model. Then, the selected checkpoint is used to test the model on the testing fold. The Cyclic learning rate [33] between $10^{-4}$ and $10^{-2}$, Adam optimizer [34], and binary cross-entropy loss were used for the enteral feeding tube dataset training or fine-tuning. All the models were trained for 100 epochs. We used Python as the programming language and PyTorch [35] as the scientific computing library to conduct the evaluation. For the ImageNet pre-trained model, we loaded the PyTorch pre-trained weights directly in to the model. The training was performed on a GPU cluster that has a combination of 120 Nividia P100 and V100 GPU cards. However, only one GPU card was used for the training at the same time.

#### Evaluation metrics

Four evaluation metrics were used in this study, namely the AUC, F1 score, accuracy, and the expected calibration error (ECE) [36]. The AUC, F1 score, and accuracy were used to evaluate models’ performance in making accurate predictions. All three metrics were bound between 0 to 1. A higher number indicated better performance. The ECE was used to measure neural network calibration error, i.e., how accurately the network estimates its prediction confidence, with a smaller value indicating a more accurate representation of its prediction confidence. A perfectly calibrated neural network has a 0 ECE.

We defined the accuracy, AUC, and F1 score following common practice. The ECE was defined as the same as [36, 37] by partitioning predictions into *M* bins and taking a weighted average of the difference of accuracy and confidence for each bin. More specifically, we first grouped all the samples into *M* interval bins according to the predicted probability. Then, let $B_m$ be the set of indices of samples whose predicted confidence falls into the interval $I_m=(\frac{m-1}{M}, \frac{m}{M}]$, $m \in M$. The ECE can be calculated as:

2 $$\begin{aligned} \text {ECE} = \sum _{m=1}^{M} \frac{|B_m|}{n} \left| \frac{1}{|B_m|} \sum _{i\in B_m} 1\cdot ({\hat{y}}^i = y^i) - \frac{1}{|B_m|} \sum _{i\in B_m} {\hat{p}}^i\right| , \end{aligned}$$

where *n* was the number of samples, ${\hat{y}}^i$ and $y^i$ were the predicted and ground-truth label for sample *i*, ${\hat{p}}^i$ was the confidence of sample *i*, $\frac{1}{|B_m|} \sum _{i\in B_m} 1\cdot ({\hat{y}}^i = y^i)$ was the accuracy of $B_m$, and $\frac{1}{|B_m|} \sum _{i\in B_m} {\hat{p}}^i$ calculated the average predicted confidence of $B_m$.

#### Model interpretation

Integrated Gradients attribution mask (IG) [38] and occlusion sensitivity testing map (OCC) [39] are used as visualization methods to understand how predictions are made by the proposed model. IG is an interpretability technique for CNN models that visualize the important features that contribute to the model’s prediction. Higher values in an IG attribution mask indicate more important features in the decision-making process. OCC is a technique for understanding which parts of an image are most important for a CNN classification. The higher values in an OCC map indicate more important areas for the image during the CNN classification procedure.

## Results

Table 1 presents the detailed evaluation result of the five models with the mean score and the 95% confidence interval of each evaluation metrics over the five trials. From the table, we can see that the proposed method has the best overall performance, which has better or comparable performance to other compared models in all settings. The *C2L* model achieves the second-best overall performance on prediction accuracy evaluation metrics but performs poorly on network calibration. The *ImageNet* model wins third place. The *No Pre-Train* model has a better performance than the *Random* model and gets the fourth place. The *Random* model performs the worst.

**Table 1:** Performance of each model (mean, $95\%$ confidence interval)

Method	AUC	F1 Score	Accuracy	ECE
	(larger is better)	(larger is better)	(larger is better)	(smaller is better)
Random	0.49, [0.45 to 0.53]	0.39, [0.35 to 0.43]	0.48, [0.44 to 0.52]	0.11, [0.10 to 0.12]
No Pre-Train	0.56, [0.50 to 0.62]	0.32, [0.23 to 0.41]	0.69, [0.67 to 0.71]	0.12, [0.10 to 0.14]
ImageNet	0.67, [0.71 to 0.63]	0.53, [0.49 to 0.57]	0.74, [0.71 to 0.77]	0.12, [0.09 to 0.15]
C2L	0.73, [0.70 to 0.76]	0.62, [0.57 to 0.67]	**0.79, [0.77 to 0.81]**	0.15, [0.13 to 0.17]
Proposed	**0.76, [0.71 to 0.81]**	**0.64, [0.58 to 0.70]**	0.78, [0.75 to 0.81]	**0.09, [0.07 to 0.11]**

The table shows that the *Random* model has a 0.49 AUC, which is essentially random guessing. The *No Pre-Train* model yields a 0.56 AUC. The *ImageNet* improves the number to 0.67. The *C2L* further improves it to 0.73. The proposed method has the highest AUC score of 0.76. For the F1 score, the *Random* model, *No Pre-Train* model, and *ImageNet* model have F1 scores that are either close to 0.5 or lower, with the worse of 0.32 for the *No Pre-Train* model. The proposed method can improve the F1 score by 100% when compared with the *No Pre-Train* model, from 0.32 to 0.64, which is also 20.75% and 3.13% higher than the *ImageNet* and *C2L* models, respectively. The *C2L* model has the highest accuracy, 79%, which is approximately 1% higher than the proposed model (78%). The *No Pre-Train* model and *ImageNet* model have 74% and 69% accuracy, respectively. The *Random* model has 49% accuracy. The *C2L* model has the highest calibration error, 0.15 ECE, which is 66.67% higher than the proposed method (0.09). Both the *No Pre-Train* model and *ImageNet* model have 0.12 ECE. The *Random* model has 0.11 ECE.

**Fig. 4:**
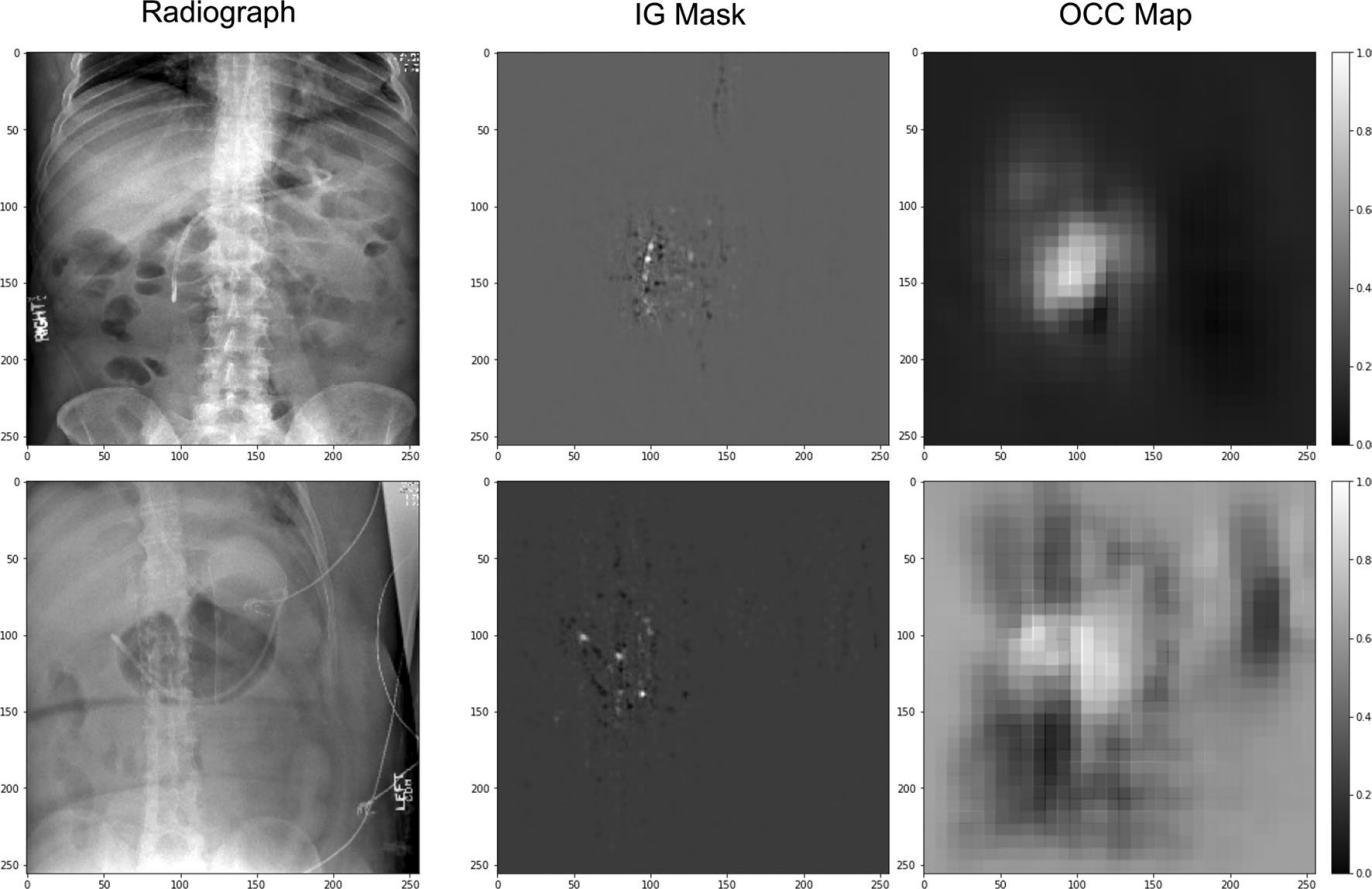
Visualization of two correct predictions

**Fig. 5:**
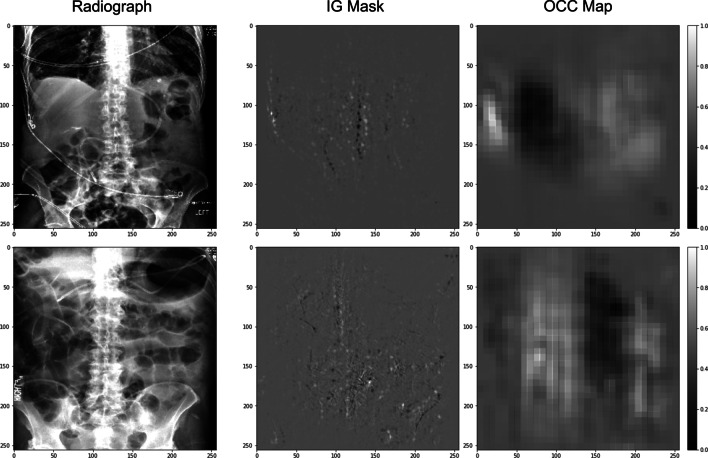
Visualization of two wrong predictions

Figures 4 and 5 show four examples of the enteral feeding tube positioning assessment results and corresponding Integrated Gradients attribution mask (IG) and occlusion sensitivity testing map (OCC). Figure 4 shows that for the correctly predicted cases, the IG and the OCC highlight the areas that critical to assessing the enteral tube positioning, while Fig. 5 shows that for the failure cases, the network was focusing on the areas that were less important to assessing the enteral tube positioning.

## Discussion

The hypothesis of our proposed method transfers natural language to image features of the architecture during the radiograph-report matching stage. The radiograph-report matching process pretrains the radiograph feature learning component parameters to the extent that it needs fewer supervised instances for the feeding tube positioning assessment task.

The result reveals that the *Random* model has an essential random performance (0.49 AUC and 48% accuracy) that sets the bottom-line performance of the enteral feeding tube positioning assessment task on this given dataset. The *No Pre-Train* improves the AUC and accuracy to 0.56 and 69%, respectively, which indicates the model did learn something about assessing the tube positioning from the training set. However, the low F1 score of the *No Pre-Train* (0.32) may suggest that the model is extremely biased to one class when making the prediction. This unexpected behavior is likely due to the small size of the training data. It is widely accepted that transfer learning helps to improve model performance on a small dataset. As expected, all three transfer learning models have a better performance than the *No Pre-Train* model. The *ImageNet* model improves the AUC to 0.67, the *C2L* model pushes the number to 0.73, and the proposed method achieved a 0.76 AUC. Though both the F1 score and accuracy are also improved by the three transfer learning models, the larger gap between the F1 score and accuracy of the *ImageNet* and *C2L* models may indicate those two models also favor one class when making the decision, especially for the *ImageNet* model.

Ideally, a predicting model should be able to reflect the uncertainty or the confidence of its prediction accurately. Otherwise, it may be problematic. For instance, given *k* predictions with average prediction confidence of *c* ($c \le 1.0$), we could expect $\approx k\times c$ correct predictions or an automatic $\approx c$% accuracy. However, the average prediction confidence often does not match the accuracy for the modern deep neural networks [40–42]. ECE is the common metric to evaluate neural network calibration error. We believe accurately estimated CNN prediction confidence is extremely important to automatic medical imaging analysis tools because an automated method that achieves high accuracy but captures prediction confidence inaccurately could lead to significant treatment errors [43]. Table 1 shows that the proposed model has the lowest ECE (0.09), which is 40% less than the *C2L* model and 25% less than the *ImageNet* model. One reasonable explanation is the precise guidance of the proposed method helps reduce the ECE on the downstream application. The proposed method is pre-trained on radiographs with radiology reports that are more relevant to enteral feeding tube positioning assessment than ImageNet pre-training, which uses natural images to pre-train the network weights. Though the *C2L* is also pre-trained on radiographs, the method uses a self-supervised strategy and provides an open ending to some degree.

By observing the correctly and wrongly predicted cases, we notice that most of the wrongly predicted cases are more challenging than the correctly predicted cases, even for human experts. For example, the first case in Fig. 5 contains other wires, which confuse the model. The IG and OCC show that the model pays more attention to those external wires when making the decision. The second case in Fig. 5 contains stronger noisy patterns, which make the images harder to read. A direct approach to improve the performance of challenging cases is to obtain more training data of challenging cases. However, this may not be easy due to the high cost of data collection. Thus, we plan to tackle this scenario from the algorithmic perspective by adding weights to challenging cases and using boosting strategies.

One limitation of this study is the lack of a standalone dataset for testing since the dataset is small. We apply a fivefold cross-testing strategy to generate a more objective testing result. Such a strategy is widely used in other specific imaging domains [44, 45]. It may be more objective than regular fivefold cross-validation because the results are based on the unseen testing set, not the validation set. A multi-site, large-scale evaluation may still be needed for further testing before using the proposed method in clinical practice.

## Conclusion

We propose a novel enteral feeding tube positioning assessment network, which can be trained using a small-scale dataset. Our evaluation results show that the proposed model has a high prediction accuracy and a more accurate estimated prediction confidence. The proposed method can be potentially used for assessing the enteral tube positioning. It also provides a strong baseline for future studies.

## Data Availability

The dataset used and/or analyzed in this study are available from the corresponding author upon reasonable request.

## Abbreviations

ANN: Artificial neural network
AUC: Area under the receiver operating characteristic curve
BERT: Bidirectional Encoder Representations from Transformers
CAD: Computer-aided diagnosis
CNN: Convolutional neural network
AD: Alzheimer’s disease
Conv: Convolutional
ECE: Expected calibration error
FC: Fully connected
GAP: Global average pooling
GWAS: Genome-wide association study
MLP: Multilayer perceptron
IG: Integrated gradients attribution
OCC: Occlusion sensitivity testing
SNP: single-nucleotide polymorphisms
